# PE_PGRS33, an Important Virulence Factor of *Mycobacterium tuberculosis* and Potential Target of Host Humoral Immune Response

**DOI:** 10.3390/cells10010161

**Published:** 2021-01-15

**Authors:** Eliza Kramarska, Flavia Squeglia, Flavio De Maio, Giovanni Delogu, Rita Berisio

**Affiliations:** 1 Institute of Biostructures and Bioimaging, IBB, CNR, 80134 Naples, Italy; eliza.kramarska@gmail.com (E.K.); flavia.squeglia@cnr.it (F.S.); 2 Dipartimento di Scienze di Laboratorio e Infettivologiche, Fondazione Policlinico Universitario “A. Gemelli”, IRCCS, 00168 Rome, Italy; demaioflavio@yahoo.it (F.D.M.); giovanni.delogu@unicatt.it (G.D.); 3 Dipartimento di Scienze biotecnologiche di base, cliniche intensivologiche e perioperatorie—Sezione di Microbiologia, Università Cattolica del Sacro Cuore, 00168 Rome, Italy; 4 Mater Olbia Hospital, 07026 Olbia, Italy

**Keywords:** vaccine, protein structure, tuberculosis, infectious disease

## Abstract

PE_PGRS proteins are surface antigens of *Mycobacterium tuberculosis* (*Mtb*) and a few other pathogenic mycobacteria. The PE_PGRS33 protein is among the most studied PE_PGRSs. It is known that the PE domain of PE_PGRS33 is required for the protein translocation through the mycobacterial cell wall, where the PGRS domain remains available for interaction with host receptors. Interaction with Toll like receptor 2 (TLR2) promotes secretion of inflammatory chemokines and cytokines, which are key in the immunopathogenesis of tuberculosis (TB). In this review, we briefly address some key challenges in the development of a TB vaccine and attempt to provide a rationale for the development of new vaccines aimed at fostering a humoral response against *Mtb*. Using PE_PGRS33 as a model for a surface-exposed antigen, we exploit the availability of current structural data using homology modeling to gather insights on the PGRS domain features. Our study suggests that the PGRS domain of PE_PGRS33 exposes four PGII sandwiches on the outer surface, which, we propose, are directly involved through their loops in the interactions with the host receptors and, as such, are promising targets for a vaccination strategy aimed at inducing a humoral response.

## 1. Introduction

Tuberculosis (TB) is still the world’s leading infectious cause of death, according to the World Health Organization (WHO) [1,2]. Its etiological agent, *Mycobacterium tuberculosis* (*Mtb*), kills approximately two million people every year and latently infects one third of the world’s population. Latency is one of the most remarkable features of TB infection, where *Mtb* establishes a dynamic equilibrium with the host immune system that lasts for lifetime, with no signs or symptoms of disease [3,4]. It is assumed that during latency, *Mtb* persists in host tissues mostly in a dormant state. Resuscitation from dormancy, which is orchestrated by a set of cell wall hydrolases [5,6,7,8,9], is a regular event in the homeostasis of *Mtb* infection that continuously replenishes the bulk of replicating bacilli after their elimination by the host immune response [10,11]. TB reactivation occurs when the equilibrium between *Mtb* and the host immune response is broken in favor of bacterial replication and tissue damage.

Active TB disease is curable with long-lasting multidrug therapeutic regimens, but the emergence of drug-resistant TB represents a major obstacle to future TB care, with important economic and social consequences [12]. Ambitious goals for better controlling the global TB epidemic can be met with the development of new drug treatments, improved diagnostics, and most importantly the availability of a new and more effective vaccine [1,2]. At present, and 100 years after its introduction, *Mycobacterium bovis* Bacille Calmette and Guérin (BCG) is the only vaccine available for TB control [13,14], though its protective activity in preventing TB in adults is variable, incomplete, and overall insufficient [15,16,17]. There is an urgent need for a new and more effective vaccine, yet poor understanding of the complex relationship between *Mtb* and the human immune system, paired with the lack of immunological correlates of protection, makes this endeavor challenging [18]. In this review, we provide a panoramic of the current *status* of vaccine development against TB and highlight the potential role of PE_PGRS proteins, a class of surface proteins with interesting immunomodulatory properties, as vaccine antigens. Given the lack of structural information on PE_PGRS proteins and the importance of structural data to understand protein function, we fill this experimental gap using homology modeling. Using PE_PGRS33 (Rv1818c) as a prototype, this review provides a basis for the unraveling of the functional properties of PE_PGRS proteins as immune modulators.

## 2. Vaccines in TB: Current Status

In the last two decades, significant progress has been made in the search for a better vaccine against TB [19,20,21,22,23,24]. Table 1 provides an overview of the main TB vaccine candidates currently in clinical development and reports their composition. TB vaccine candidates can be grouped into two main categories: whole cell-derived vaccines and subunit vaccines. The first group can be subcategorized into live attenuated mycobacteria and killed or fractionated whole mycobacteria. The second one can be broken down into protein subunit vaccines and recombinant viral-vectored vaccines (Table 1).

The main advantage of a whole cell-derived vaccine is the presence of different antigenic components and the possibility to stimulate a broader and diverse immune response than subunit vaccines without the addition of adjuvants, since the mycobacterial cell wall serves as a potent activator of innate immune response (Table 1). Conversely, subunit vaccines or recombinant viral or bacterial vectored vaccines target one or few antigens, selected because of their immunogenicity, such as Ag85A, Ag85B, ESAT-6, CFP-10, and PPE18 [18,25]. All the new vaccines that already entered clinical trials and most of those in the preclinical stage aim at inducing a Th1-type cell response against one or more *Mtb* antigens, to promote early and rapid recruitment of “protective” T cells secreting the Interferon-γ (IFN-γ) at the site of *Mtb* infection [18,26]. However, the results obtained with the phase IIb clinical trial testing a prime-boost strategy with BCG and MVA85A highlighted the complexity and challenges associated with this strategy [27]. Mounting evidence collected in many experimental and clinical studies indicates that Th1 responses are necessary but not sufficient to mediate protection against *Mtb* and that other functions of the immune responses, perhaps including the somehow neglected humoral arm, are involved in the process. Moreover, certain types of cell-mediated immunity against *Mtb* are associated with the immunopathology rather than protection, and identifying the T cell immune phenotypes involved, or their *Mtb* antigen targets, is a still unsolved task [28,29,30,31]. This is of paramount importance considering that the immunodominant antigens are highly conserved and result in purifying selection in *Mtb*, suggesting that triggering powerful T cell responses in the human host is part of the *Mtb* survival strategy [32,33]. In a granuloma, the T cell responses against immunodominant *Mtb* antigens may promote T cell exhaustion, which impairs host immunity while favoring *Mtb* replication and tissue damage [34]. Designing a new vaccine against TB requires a fine understanding of these immunological processes since eliciting powerful T cell responses against strongly immunogenic and highly expressed and conserved *Mtb* antigens may be potentially deleterious [26].

The role of the humoral response has been questioned for a long time, though recent studies have brought new and compelling evidence for the role of antibodies in *Mtb* infections. BCG vaccination is known to induce antibodies, primarily against capsular polysaccharides as arabinomannan and α-glucans [48]. IgG directed against arabinomannan obtained from asymptomatic but not diseased patients is protective in relevant experimental models [49], and immunization with an arabinomannan–protein conjugative vaccine elicits levels of protection in animal experiments [50]. Hence, surface antigens may be the target of a potentially protective immune response against TB mediated by antibodies, with capsular antigens being important in this process [51]. Antibodies obtained from latent TB subjects are more effective in inhibiting *Mtb* growth compared to antibodies obtained from active TB patients, thanks to the ability of the former to boost macrophages to kill intracellular *Mtb* [52]. A group of household contacts of TB patients who did not convert to TST or IGRA (termed “resisters”) had antibodies against secreted *Mtb* antigens as Ag85A and ESAT-6/CFP10, suggesting a potential role of the humoral response in “resisting” infection or at least in preventing the development of overt disease [53]. Hence, mounting evidence supports the role of antibodies in protection against TB, although the functional differences in their efficacy observed between groups of different *Mtb* infected subjects requires further scrutiny [54]. Current knowledge indicates that the classical targets of a protective humoral response are surface or secreted antigens that exert a functional role in microbial pathogenesis.

Designing new vaccines against TB aimed at eliciting protective antibodies requires a better understanding of the mycobacterial surface antigens, with emphasis on the protein antigens that localize on the outer surface and may be available in the mycobacterial capsule. Among these antigens are proteins belonging to the PE and PPE families, which are unique for virulent mycobacterial species. Some of these proteins have been shown to interact with host components and, as such, are implicated in TB pathogenesis. In this review, we build on structural and functional data to argue that a protein belonging to the PE_PGRS subfamily, PE_PGRS33, may serve as a target for a protective humoral response against *Mtb*, raising the possibility for new vaccination strategies against TB that target unique surface antigens.

## 3. The PE_PGRS Family of Surface Proteins

Nearly 10% of the *Mtb* genome coding potential is occupied by unique and peculiar *pe* and *ppe* genes, which code for the corresponding protein families [55,56]. PE and PPE protein families take their names from conserved motifs in their N-terminal regions [57]: specifically in residues 7–8 (proline–glutamic acid) for PE and 7–9 (proline–proline–glutamic acid) for PPE [56]. From almost 100 known representatives of the PE protein family [58], we can distinguish three subfamilies, including (i) the PE-only subfamily (< 100 aa in length), which is typically associated with a PPE protein to form a heterodimer; (ii) the PE_unique subfamily, which presents unique amino acid sequences of various length at the protein C-terminal side [59], such as the LipY triacylglycerol lipase, embedding an α/β hydrolase responsible for the hydrolysis of intracellular and extracellular triacylglycerol (TAG); (iii) the PE_PGRS subfamily, which contains numerous glycine-rich sequences (GGA-GGX repeats) [60,61]. Over 60% of the known PE proteins belong to the PE_PGRS subfamily [58].

PE_PGRS localize on the mycobacterial surface [60,62,63,64], with its transport through the mycobacterial membrane carried out by the ESX5 system, a type VII secretion system specialized in the secretion of mycobacterial proteins [57,65,66,67]. Exact roles of PE_PGRS remain unknown, but they have been shown to play a role in *Mtb* virulence, in particular in the chronic/persistent phase of the infection [68]. During this phase, PE_PGRS proteins accumulate in the necrotic and caseous granulomas [69,70], where they can promote inflammation by directly interacting with Toll-like receptors (TLRs) through the PGRS domain [71,72]. Specifically, it has been shown that the PGRS domain of PE_PGRS5 (Rv0297) leads to cell death via TLR-4 dependent endoplasmic reticulum (ER) stress [73]. Similarly, PE_PGRS33 [71,74], PE_PGRS11, and PE_PGRS17 [75] are able to interact with TLR-2, whereas PE_PGRS11 and PE_PGRS17 affect the maturation of human dendritic cells (DCs) and their capacity to activate robust proliferation and production of IFN-γ and IL-5 in CD4+ T cells [75]. PE_PGRS proteins and, in general, PE and PPE proteins are processed and presented by DCs to elicit robust T cell responses, with the MHC-I and MHC-II epitopes detected in the highly conserved PE and PPE N-terminal domains, while T cell epitopes are poorly represented in the PGRS domain [61]. Strong T cell responses are directed against the epitopes in the PE domains during *Mtb* infection [76].

Antibodies against PE_PGRS proteins and specifically targeting their PGRS domain have been detected in *Mtb*-infected animals and TB patients [77,78,79]. It remains to be determined what are the functional roles of these antibodies and whether these can interfere with the PE_PGRS–host cell receptor interaction. Opsonization may promote *Mtb* killing by activated macrophages; in this case, antibodies may exert their activity since the early steps of *Mtb* infection. Neutralization of surface antigens may be of relevance also during the late stage of TB pathogenesis, by blocking the interaction of PE_PGRSs with TLRs or other host receptors, thereby reducing the inflammation and tissue damage that is the hallmark of TB disease [80]. Unfortunately, suitable experimental models are needed to properly assess the protective mechanisms of antibodies targeting PE_PGRSs.

## 4. Structural Features of PE_PGRS33, the PE_PGRS Prototype

**Figure 1:**
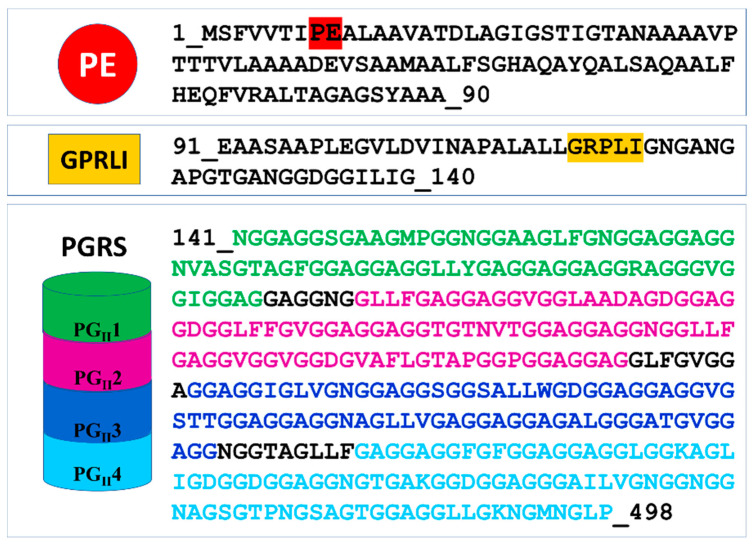
Domain organization and sequence of PE_PGRS33. In the PE domain, the conserved PE motif is colored red. In the PGRS domain, sequences of the four PG_II_ sandwich motifs are colored green, purple, blue, and cyan.

Knowing the three-dimensional structure of an antigen provides important insights into the understanding of the molecular nature of host–pathogen interactions and of the key epitopes that may serve as a target for the host antibody response. Although structural data on PE_PGRS proteins are not available, insightful information can be obtained by modeling techniques, learning from homologous proteins. Among PE_PGRS proteins, PE_PGRS33 is one of the best studied for its interaction with the immune system and has been considered a model for PE_PGRSs. As such, its putative role as a vaccine candidate is worth being investigated [81]. PE_PGRS33 is a large protein of 498 residues, with a modular architecture. A search in the PFAM database only identifies a PE domain at the N-terminal region of the protein, a small domain (residues 1–93) that is distributed nearly exclusively in actinobacteria, with only one exception for *Tulasnella calospora*, a genus of patch-forming fungi in the *Tulasnellaceae* family. A conserved linker GRLPI domain (l-GRPLI), likely acting as a transmembrane anchor, connects the PE domain to a distinctive region, not predicted by PFAM, and characterized by multiple repeats containing the GGA-GGX motif interspersed with unique sequences, and commonly denoted as PGRS domain (Figure 1).

### 4.1. The PE Domain

The PE domain takes its name from the conserved Pro–Glu (PE) amino acids at its N-terminus (residues 7–8) [55] Figure 1). This domain is responsible for PE_PGRS33 translocation via ESX5 and cell wall localization, with a significant role of 30 amino acids on its N-terminus [82]. A search in the PFAM database shows that the PE family is a member of clan EsxAB (CL0352), which also includes the more common PPE family and the WXG100 family, including the well-known antigen ESAT-6 (6 kDa early secreted antigenic target) and CFP-10 (10 kDa culture filtrate protein) in *Mtb* or EsxA (ESAT-6-like extracellularly secreted protein A) and EsxB in *Staphylococcus aureus*.

**Figure 2:**
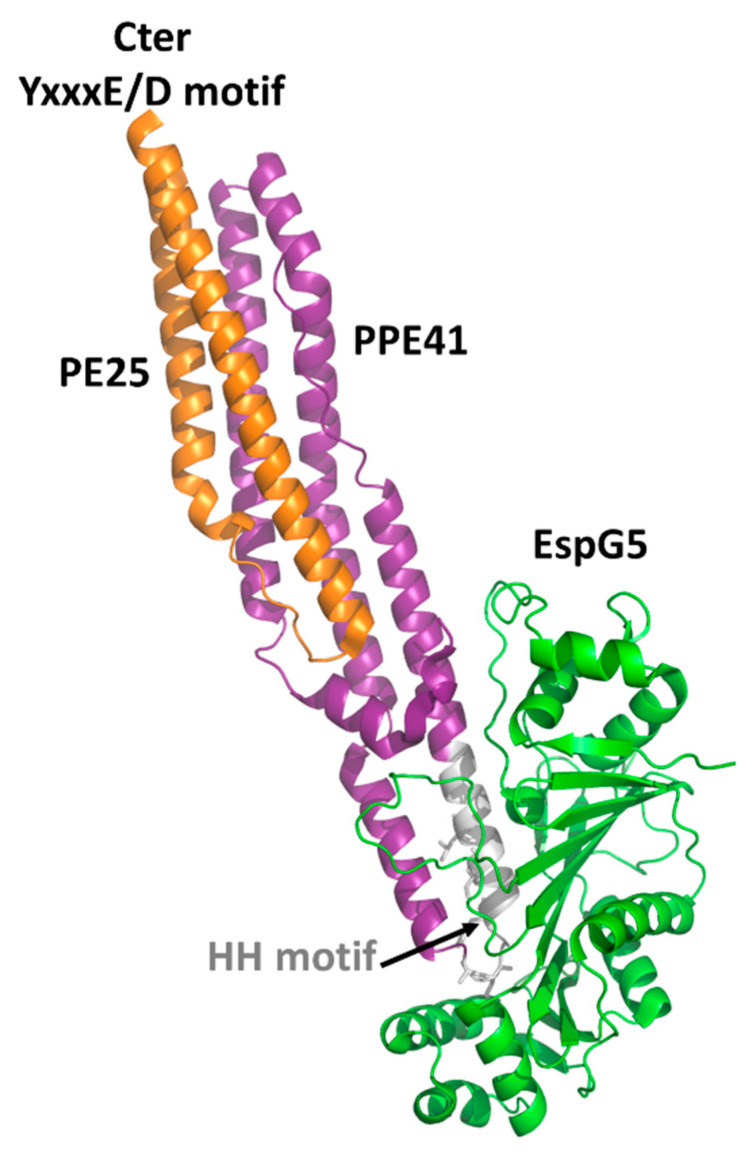
Cartoon representation of the crystal structure of PE25-PPE41 (orange/purple) ternary complex with the chaperone EspG5 (green). The signature ESX secretory motif YxxxE/D is located at the C-terminal side of PE25 (orange), whereas the EspG5 binding region is located on the HH motif of PPE41 (gray) [83].

Crystal structures have been reported for the two PE domains PE8 and PE25, in both cases forming a heterocomplex with PPE partners (Table 2). In all structures, PE and PPE interact via a hydrophobic interface forming a four-helix bundle formed by two α-helices from the PE and two α-helices from the PPE module (Figure 2). The crystal structure of the ESX-5-secreted PE25–PPE41 heterodimer in complex with the ESX-5-encoded cytoplasmic chaperone EspG5 shows that EspG5 binds to a highly conserved hydrophobic chaperone-binding sequence on PPE, named as the hh motif [83] (Figure 2).

By binding PPE, EspG5 protects the aggregation-prone hh motif on PPE proteins and keeps the dimers in a secretion-competent state. Consistently, point mutations of this conserved hh motif affect protein secretion [83]. Both in the cases of PE25–PPE41 and PE8–PPE15, the binding of EspG5 chaperone does not cause conformational changes in the heterodimers. The two ternary complexes present highly similar structures. A superposition of their structures using DALI produces root mean square deviations (rmsd), computed on the backbone atoms of PE, PPE, and EspG5 chains, of 1.1 Å, 2.1 Å, and 0.5 Å, respectively. Importantly, EspG5 binds the PE–PPE dimers at a location that does not interfere with the signature ESX secretory motif YxxxD/E at the C-terminal side of PE proteins [87] (Figure 2).

**Figure 3:**
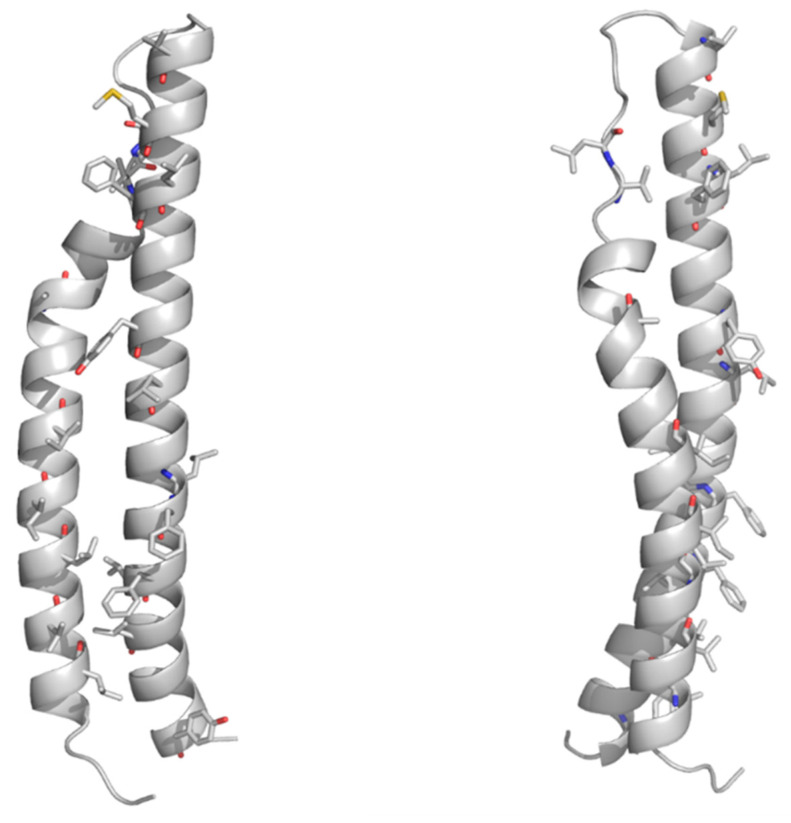
Cartoon representations of the homology model of the PE domain of PE_PGRS33. Front and side views are reported on left and right sides, respectively. The model was computed with MODELLER using the structure of the PE25 domain from a type VII secretion system of *Mycobacterium tuberculosis* (*Mtb*) as a template (PDB core 4w4k, sequence identity 37%). Hydrophobic and aromatic residues are drawn in stick representation.

As mentioned above, no structural information on the PE domains from PE_PGRS proteins is hitherto known. Neither it is known whether PE_PGRS proteins strictly require a PPE-like domain, as in the case of PE proteins in Table 2. Here, we fill this structural gap by adopting homology modeling. The best template was identified by HHPRED as the PE domain the ESX-5-secreted PE8 (PDB code 5xsf, sequence identity 45.8%) and the homology model built using MODELLER [88,89,90,91]. As a result, the homology model of the PE domain of PE_PGRS33 shows that all hydrophobic/aromatic residues are located on one side of the molecule (Figure 3). This feature, also observed for the PE domains of PE/PPE complexes, suggests that either the PE domain of PE_PGRS33 forms homodimers or it is prone to interact with another protein to form a heterodimer. It is hitherto not clear whether PE_PGRS proteins require a protein partner [59,86], as in the case of PE/PPE proteins. Indeed, pe_pgrs genes are expressed as single operons. Also, the PE-unique LipY protein does not require a partner to be secreted [92]. These findings suggest that PE_PGRSs can be stable on their own, albeit being endowed with prone-to-interact PE domains for their functions.

**Figure 4:**
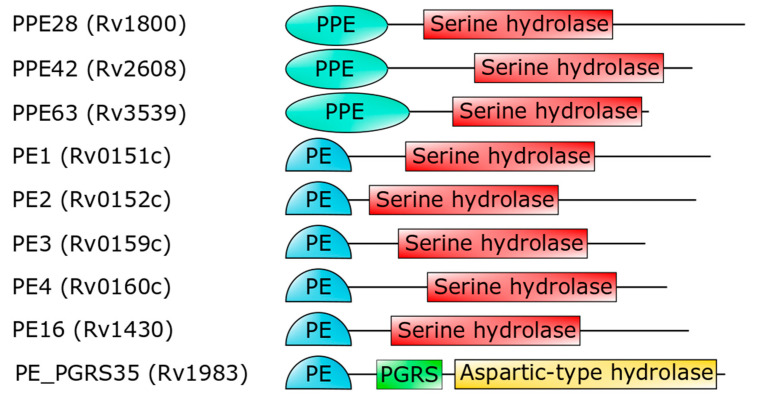
Domain architecture of PE- or PPE-containing *Mtb* proteins (strain H37Rv), which are predicted to embed a serine hydrolase domain [93]. The last hydrolase, PE_PGRS35, is the *Mtb* homolog of *M. marinum* PecA [92].

Importantly, the PE domain of PE_PGRS33 is required for the protein translocation through the mycobacterial cell wall [63,82,88,90,91]. Once exerted this role, the PE domain is cleaved from the rest of the molecule, leaving the functional PGRS domain floating on the mycomembrane [59]. Therefore, it is tempting to surmise that some hydrolases may recognize PE_PGRS33 through its PE domain. *Mtb* encodes for a number of PE- and PPE-containing serine α/β hydrolases, which are possible candidates as PE_PGRS33 hydrolases [93] (Figure 4). In addition to these, a PE_PGRS aspartic-type endopeptidase, denoted as PecA in *M. marinum* and PE_PGRS35 in *Mtb*, is known to cleave the lipase LipY [92]. More investigations are needed to verify which hydrolase is responsible for PE cleavage of PE_PGRS33 and if more hydrolases cleave specific PE_PGRS proteins.

### 4.2. PGRS Domain Contains Multiple PG_II_ Modules

In PE_PGRS proteins, the PGRS domain can vary in size from tens to almost 1800 amino acid residues. Its main feature is the presence of multiple repeats containing the GGA-GGX motif interspersed with unique sequences [94]. It has been shown that PGRS domains are available on the mycobacterial surface and can directly interact with host components, as TLR2 receptors [60,74,95]. To date, the structure of the PGRS domain remains unknown and should be implemented in experimental data.

The lack of structural data on PGRS domains makes the understanding of the role of these domains a hard task. However, a high sequence identity of the C-terminal part of the PGRS domain of PE_PGRS33 exists with the PG_II_ domain of snow flea antifreeze protein sfAFP from *Hypogastrura harveyi* (sequence identity 60% with residues 406–486). Therefore, we performed homology modeling based on the target–template alignment using ProMod3 and the structure of sfAFP as a template (PDB code 2pne). The alignment of the sequence of this C-terminal PG_II_ module against the entire PGRS region identifies further three modules with the same pattern and sequence identities ranging between 63.0% and 53.9% (Figure S1). This analysis shows that the PGRS domain of PE_PGRS33 is formed by four PG_II_ domains, denoted here as PG_II_1, PG_II_2, PG_II_3, and PG_II_4, all with similar structural features.

**Figure 5:**
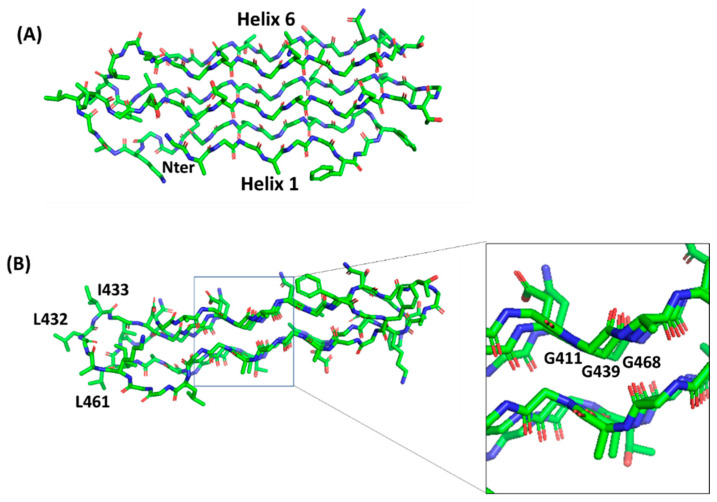
Stick representation of the homology model of the PG_II_ sandwich domain PG_II_4, computed using the structure of sfAFP as a template (pdb code 2pne). The front view (**A**) shows the six PG_II_ helices, whereas the side view (**B**) shows the localization of hydrophobic residues (e.g., L432, I433, L461) on the lateral loops. The inset shows glycine residues pointing inside and stabilizing the tightly packed PG_II_ helices.

Polyglycine conformations, such as PG_II_, are the most flexible ones because the lack of side chains in glycine removes steric hindrances. Consequently, extended regions of the Ramachandran plot are allowed for glycine residues, which can virtually assume any *ψ* angle [96]. Typical of PG_II_ conformation, each glycine-rich triplet folds into a left-handed, elongated helix with a pitch (rise per turn) of 9.2 Å. This conformation resembles that observed for the polyproline type II (PP_II_) helices found in collagen [97,98]. In the PG_II_ sandwich, six antiparallel PG_II_ helices are stacked in two antiparallel groups, with three to four triplets spanning the PG_II_ domain length (Figure 5). The organization of the PGRS region in PG_II_ domains explains the high abundance of glycine residues in these domains. Glycine residues are always pointing inwards, in positions where only glycine could be sterically allowed (Figure 5).

**Figure 6:**
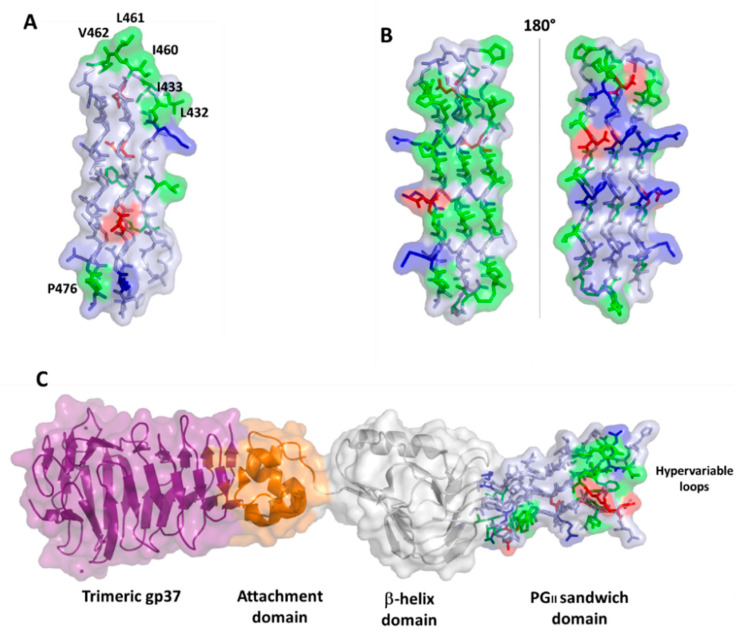
Surface and stick representations of PG_II_ domains in (**A**) PE_PGRS33 (domain PG_II_4); (**B**) antifreeze protein sfAFP (pdb code 2pne) in two 180° views; and (**C**) *Salmonella* bacteriophage S16 long tail fiber (pdb code 6F45). In this panel, the PG_II_ domain is located at the C-terminal side of the protein (stick and surface representation). Adjacent domains are drawn in surface and cartoon representations. In all panels, the color code used for PG_II_ residues is red for negative, blue for positive, green for hydrophobic, and light blue for polar residues.

A comparison with the structure of the PE_PGRS33 PG_II_ domains with that of the antifreeze protein sfAFP highlights different surface characteristics, albeit presenting the same fold, likely due to completely different functions of PG_II_ domains in the two proteins (Figure 6). In PE_PGRS33, hydrophobic residues of PG_II_ domains are located mainly on loop regions (Figure 5B and Figure 6A), likely accounting for a role of these residues in host recognition. Consistently, as will be shown later, removal of consecutive residues belonging to PG_II_ domains of PE_PGRS33 (alleles from 48 to 52, Table 3) results in weaker immunostimulatory activity, in terms of reduced TNF-α [72,74]. By contrast, hydrophobic residues are mostly located on one side of the PG_II_ structure of sfAFP (Figure 6B) [99]. The accumulation of hydrophobic residues on one side of the sandwich builds a module with one hydrophilic face and one hydrophobic face, and the flat hydrophobic face is supposed to interact tightly with the highly ordered water molecules found at the surface of an ice crystal [99]. In this respect, the flatness that characterizes this domain is functional for its tight association with the ice surface [99]. Interestingly, a PG_II_ sandwich was also observed in the *Salmonella* bacteriophage S16 long tail fiber. In this case, this PG_II_ sandwich domain plays a role in the interactions of the phage with its host, with its exposed (hydrophobic) loops being determinants of host binding. Therefore, similar to PG_II_ of PE_PGRS33, the glycine-rich core of the PG_II_ sandwich of S16 long tail fiber exposes hypervariable β-turn loops that determine receptor specificity (Figure 6C).

In contrast to the antifreeze protein sfAFP, it is likely that in the case of both PE_PGRS33 and the bacteriophage S16 long tail fiber, the flatness of the PG_II_ sandwich is useful to allow recognition loops to be closely spaced. In both cases, these loops evolve rapidly, as confirmed by their hypervariable nature, in a similar manner as observed for the three hypervariable complementarity-determining regions (CDRs) of immunoglobulins [100,101]. As in the case of S16 tail fiber, the PG_II_ loops of PE_PGRS33 are likely exposed to the host and, as such, the principal targets of antibodies.

## 5. PE_PGRS33 as a Promising Target of the Humoral Response

**Figure 7:**
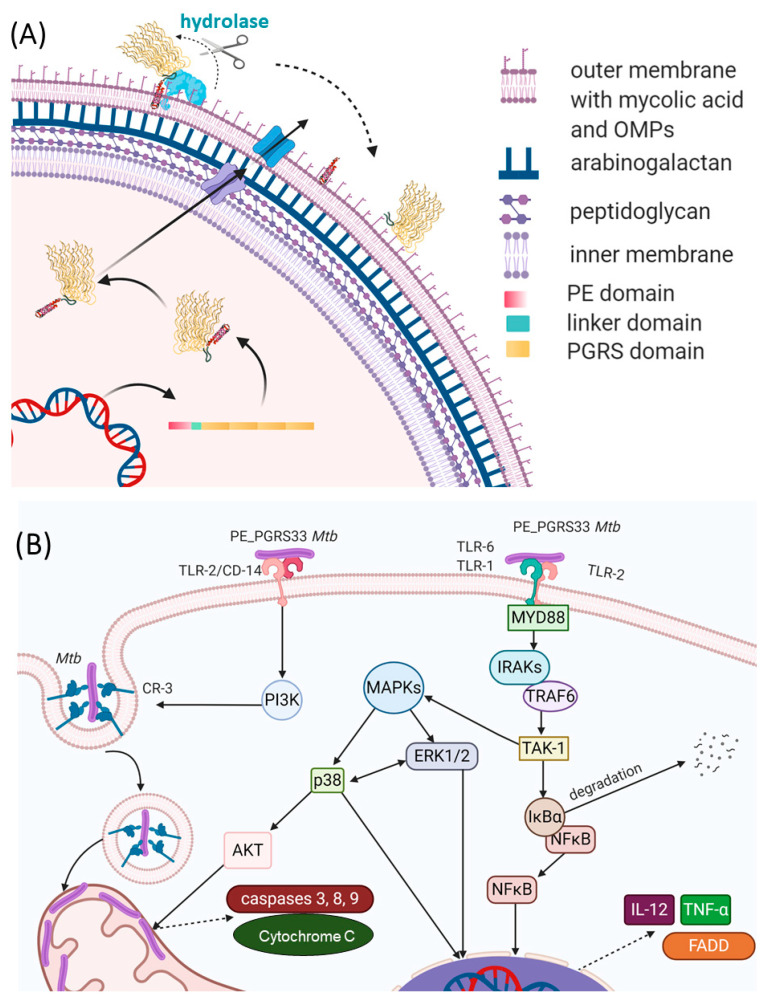
Schematic representations of (**A**) the PE_PGRS33 path to the mycobacterial membrane and (**B**) immune responses to PE_PGRS33 by the host.

PE_PGRS33 promotes cell death and increases mycobacterial survival in macrophages, as demonstrated by heterologous expression in *Mycobacterium smegmatis (Ms)*, in a process mainly governed by its PGRS domain [74,102,103]. Being localized on the outer surface of the *Mtb* cell wall, PE_PGRS33 is in a position that results in the exposure to the *milieu* and in the capacity to interact with the host [95] (Figure 7A). Consistently, PE_PGRS33 was shown to specifically interact with TLR-2 [71,74], though it remains to be determined whether TLR2 requires heterodimerization with TLR1 or TLR6 to properly bind PE_PGRS33, and the role of coreceptors as CD14 or CD36 as well.

The binding of PE_PGRS33 with TLR-2 on macrophages can activate two different intracellular pathways. Activation of the Myd88 pathway triggers the expression of genes coding pro-inflammatory chemokines and cytokines in an NFkB-dependent mechanism. Secretion of tumor necrosis factor-α (TNF-α) promotes cell necrosis and inflammation, which increases the survival of *Mtb* inside the host [74] (Figure 7B). Activation of the PI3K pathway triggers the inside-out signaling pathway, which enhances *Mtb* internalization in host cells while dampening macrophage antimicrobial responses [71]. Interestingly, a polyclonal antiserum raised against native PE_PGRS33 was shown to inhibit *Mtb* entry into macrophages, without affecting the entry of the *Mtb* Δpe_pgrs33 strain [80]. This same Δpe_pgrs33 *Mtb* strain has impaired capacity to enter macrophages, likely due to the lack of interaction with TLR-2 [71,74]. Mice immunized with recombinant PE_PGRS33 were able to combat recombinant *M. smegmatis* overexpressing PE_PGRS33 in vivo [80]. Hence, PE_PGRS33/TLR2 interaction promotes inflammation and tissue damage while favoring *Mtb* replication in the host lesions, raising PE_PGRS33 as an important *Mtb* virulence factor [72]. This experimental evidence provides functional clues to the hypothesis that PE_PGRS33 may serve as a vaccine antigen candidate against TB [81,104,105]. Antibodies binding PE_PGRS33 on the *Mtb* surface may neutralize the binding with TLR2, turning off a pathogenetic pathway that promotes TB disease. PE_PGRS33-specific antibodies may also opsonize *Mtb*, prompting a more efficient uptake and killing by activated macrophages.

*Mtb*-infected and BCG-vaccinated subjects make antibodies against PE_PGRS33, with the key epitopes located mainly in the PGRS domain [104]. Most of the experimental evidence gathered so far has relied on the use of denatured recombinant PE_PGRS33 [80], making it difficult to identify the relevant epitopes responsible for the interaction with host receptors and potential targets of a protective humoral response.

Conversely, as outlined in this review, structural features of the PGRS portion of PE_PGRS33 well account for the role of this protein as a target of antibodies. The organization of PGRS in PG_II_ sandwich modules crossing the external membrane allows the protein to efficiently expose loops for epitope recognition (Figure 7). The identification of these epitopes and the fine characterization at the atomic level of the interaction between TLR2 and PE_PGRS33 would be very useful to tailor effective immunization strategies. Altogether, these studies highlight the potential use of PE_PGRS33 as a target of a neutralizing humoral response against TB [80].

## 6. PGRS PG_II_ Sandwich Structure Tolerates Polymorphism

Despite early evidence indicating PE_PGRSs as variable surface proteins responsible for *Mtb* antigenic variability and immune evasion [55,62], more recent reports show that most *pe_pgrs* genes are highly conserved and result in purifying selection [61]. The most relevant polymorphisms in *pe_pgrs*33 are indels of variable size occurring in the PGRS domain. Table 3 summarizes the most frequent alleles identified in large collections of *Mtb* clinical strains. These *pe_pgrs*33 alleles are conserved in the *Mtb* phylogeographic lineages and clades, indicating a clustering of specific alleles during *Mtb* evolution [72], while no clustering has been observed in drug-resistant strains [68]. The insertions detected in these alleles usually involve DNA sequences coding three amino acids, two triplets (GGX GGX) and up to 11 amino acids for allele 52. Deletions are more frequently detected and usually consist of regions coding one or more GGX triplets and may involve sequences coding up to 30, 48, or 90 amino acids. The functional consequences of some of these polymorphisms have been investigated in in vitro and in vivo models of *Mtb* infection. Some *pe_pgrs*33 alleles with large deletions appear to be associated with noncavitary TB or extrapulmonary TB [74,106]. Others have different immunoregulatory properties [74] or differential ability to bind TLR2, which may also affect the entry of *Mtb* in macrophages [72]. Although the impact of *pe_pgrs*33 polymorphisms on TB pathogenesis requires further scrutiny, it transpires that large indels do not lead to a complete loss of protein function [72].

The observation that even the *pe_pgrs*33 alleles showing large deletions are clustered with certain *Mtb* clades, coupled with the fact that *Mtb* transmission occurs only through the aerogenic route from patients with active pulmonary TB, indicates that these polymorphic alleles do not significantly impact *Mtb* fitness. This is apparently in contrast with the finding that *pe_pgrs*33 shows a substitution rate ratio (dN/dS) of 0.7, suggesting that a biological pressure acts on *Mtb* to prevent mutations that may impair PE_PGRS33 protein function [72]. We propose that the organization of the PGRS domain in PG_II_ sandwich modules, consisting of tightly packed helices, each made of three or four turns with three residues per turn (the GGX triplets), provides a plastic structure that can tolerate large indels while maintaining proper localization of the unique amino acids that are found in the loops between the helices. PG_II_ sandwiches are typically stabilized by hydrogen bonds involving backbone atoms of glycine residues (N—H-O and C—H-O). Therefore, glycine residues are the sole residues that cannot mutate, as their mutation to any other residues would strongly affect the structural integrity of the PG_II_ sandwich. On the other hand, mutations of non-glycine residues are predicted to hardly contribute to structural stability, as they point toward the external part of the molecule and are not involved in stabilizing intramolecular interactions. Similarly, insertions or deletions of entire triplets, as observed in most frequent alleles (Table 3), are likely to poorly affect the PG_II_ sandwich structural features. Therefore, the polymorphisms observed in the PGRS domain find their rationale in the structural features of PG_II_ sandwich domains. However, while keeping the PG_II_ structural features, the deletion of entire PG_II_ helices in PG_II_ sandwiches 1, 2, 3, and 4 may result in weaker immunostimulatory activity (reduced TNF-α) as observed in alleles 48–52 (Table 3), suggesting the presence of key protein domains with functional properties in these deleted regions, e.g., a domain that affects interaction with TLR2.

In summary, the observations made and results obtained so far suggest that (a) the PGRS domains of PE_PGRS33 expose four PG_II_ sandwiches on the outer surface; (b) these PG_II_ sandwiches are directly involved through their loops in the interactions with host receptors as TLR2; (c) the sequences encoding these regions result in purifying selection in *Mtb*, indicating a key role of PG_II_ domains in *Mtb* survival and TB transmission; and (d) insertions and deletions in the region encoding the PG_II_ domains may have an impact on the immunomodulating activity of PE_PGRS33, as assessed in in vitro and in vivo models. Hence, a strategy aimed at blocking or interfering with the PE_PGRS33–host cell receptor interaction might be useful to block the key steps of TB pathogenesis. As such, a vaccine formulation aimed at eliciting antibodies targeting the surface-exposed loops of PG_II_ domains of PE_PGRS33 may serve to curb inflammatory processes and prevent or reduce the risk of a drift toward necrosis and mounting tissue immunopathology, which is the hallmark of active TB disease.

**Table 3:** Most frequent alleles identified in large collections of *Mtb* clinical strains. PG_II_ modules of PE_PGRS33 are defined as indicated in Figure S1.

Allele *	Polymorphism *	Amino Acid Variation	Position (aa)	PG_II_	Clade/ Lineage	Functional Notes	Clinical Notes
allele 1 (allele 11) [72]	Wild type	-	-	-	4 LAM/ Haarlem		Associated with cavitary TB
allele 26 (allele 3) [72]	S4 D14 I4	−28 Gly X +1 Gly Ala	239 338–498 413	3, 4, 5	1 EAI	Impaired entry in macrophages compared to wt allele. Hypervirulent in mice	Associated with noncavitary TB
allele 45 ^1^ (allele 5) [72]	S22 ^a^ S4 I4	Gly → Ser - +1 Gly Ala	233 239 413	2, 4	Animal lineage		-
allele 461 (allele 6) [72]	D2 S4 I4	−4 Gly X 717 +1 Gly Ala	140–163 239 413	1, 4	1 EAI		-
allele 471 (allele 18) [72]	S4 D19 ^b^ I4	- −3 Gly X +1 Gly Ala	239 257–270 413	2, 4	3 Delhi/CAS		-
allele 481 (ΔG184-G213) [74]	D20 ^c^	−30 aa	184–213	1, 2	-	Weaker immunostimulatory activity (reduced TNF-α)	-
allele 491 (ΔL237-G327) [74]	D21 ^d^	−91 aa	237–327	2, 3	-		-
allele 501 (ΔL372-A403) [74]	D22 ^e^	−32 aa	372–403	4	-		-
allele 511 (ΔG196-D243) [74]	D22 ^f^	−48 aa	196–243	1, 2	-		-
allele 521 (SA440) [74]	ID2 ^g^	−36 aa +11 aa	236–271 272	2	-		-
allele 22 (SA455) [74]	I1 S4 I4	+2 Gly Ala Gly - +1 Gly Ala	199 239 413	1, 4	-		Associated with cavitary TB
allele 40	D8	−47 aa	213–260	2	-	-	Associated with non-cavitary TB
allele 531 (33260) [71]	D23 ^h^	−238 aa	260–498	2, 3, 4, 5	-	Sufficient to complement the *Mtb* Δ33 mutant in the in vitro macrophage invasion assay	-
allele 541 (Group 2) [106]	S4 I6 ^i^	- + Ala Gly	408	4	-	-	Associated with extrapulmonary TB

* According to allele sequential number and polymorphism description of Talarico et al. [107]. ^1^not described in [107]. In column 2, letters from a to i indicate: ^a^, nsSNP, position 697; ^b^, deletion of 42bp, position 772–813; ^c^, deletion of 90bp, position 552–639; ^d^, deletion of 273bp, position 711–981; ^e^, deletion of 96bp, position 1116–1209; ^f^, deletion of 144bp, position 588–729; ^g^, deletion of 107bp, position 710–816 and insertion of 32bp in position 817; ^h^, deletion of 714bp, position 780–1494; ^i^, insertion of 9bp, position 1224.

## 7. Concluding Remarks

Vaccine development against TB has been dominated by strategies aimed at the elicitation of robust T cell responses, while antibody-based strategies have been neglected due also to the limited number and poor knowledge of *Mtb* surface antigens that are the natural targets of protective humoral responses. In this work, we provide a brief summary of the main challenges associated with the development of a new vaccine against TB and highlight the importance of a structure/function view to investigate the vaccine potential of a unique class of *Mtb* surface antigens, the PE_PGRS proteins. Among these, PE_PGRS33 can be considered a prototype protein of the family for the availability of experimental data implicating the PGRS domain in the interaction with host receptors. A vaccination strategy aimed at eliciting a protective humoral response shall ideally target secreted or surface-exposed antigens and/or virulence factors that play a key role in microbial pathogenesis. PE_PGRS33 is such a candidate, although the lack of structural data on the peculiar PGRS domain has so far hampered a fine understanding of the proper localization of this protein on the mycomembrane. For years, the glycine-rich PGRS domain has been thought to be highly flexible or unfolded, due to the conformational properties of glycine residues. Using homology modeling, we show that the PGRS portion of PE_PGRS33 is well structured and contains four compact subdomains, named as PG_II_ sandwiches, each consisting of six left-handed PP-II helices stacked in two antiparallel groups. These sandwich domains were previously observed in antifreeze proteins, a class of macromolecules critical to the existence of life at sub-zero temperatures, and in *Salmonella* phage S16 adhesin. Similar to S16 adhesin, the flat structure of the PG_II_ sandwich domains of PE_PGRS33 may be functional to projecting surface-exposed loops on one side of the molecule, like antigenic loops. Overall, the structural considerations collected here for PGRS domains also allow for the interpretation of the typical polymorphism observed for PGRS domains, since the PG_II_ sandwich domain is able to tolerate modifications, insertions, and deletions, without destabilization. A peculiarity of PG_II_ sandwiches is that they are stabilized solely by backbone hydrogen bonds (N—H-O and C—H-O) involving glycine residues. This feature, with glycine residues pointing inside the fold, where no other residues would fit, explains the high frequency of glycine residues in PGRS domains.

Therefore, the unique sequences in the PGRS domain coding the surface-exposed loops are likely to be responsible for the interaction with host receptors (e.g., TLR2 for PE_PGRS33) and the ideal targets for antibodies with protective potential.

In conclusion, the data examined here provide the structural basis for both the antigenic properties of PGRS domains and of their typical polymorphism. Targeting the surface-exposed loops emerging from the PG_II_ sandwich domains with a humoral response is an out-of-the-box approach to develop new vaccines against TB, taking into account the exposure of multiple copies of PG_II_ sandwiches on the mycobacterial outer membrane.

One important aspect to address concerns the identification of an effective vaccination strategy that can elicit strong, robust, and specific antibody responses against the surface-exposed loops. Inclusion of recombinant PE_PGRS33 purified under native conditions in already available vaccine platforms, such as BCG or new vaccines against TB, may be a strategy that can be useful to pursue [80]. Immunization with BCG strains overexpressing PE_PGRS33 or with extracellular membrane vesicles [108,109] enriched in PE_PGRS33 may serve to elicit antibody responses against protein domains presenting the appropriate structure to elicit functionally active antibodies capable of neutralizing PE_PGRS33 and/or of opsonizing *Mtb*. The fact that *Mtb* expresses more than 50 PE_PGRS proteins on the mycobacterial surface, with structural features similar to those of PE_PGRS33, and that these proteins play unique roles in the *Mtb* infection process and immunopathogenesis of TB, underscores the potential impact that a vaccination strategy targeting PE_PGRS proteins may have in the control of TB.

## Figures and Tables

**Table 1:** TB Vaccine candidates in clinical trials.

TB Vaccine Candidate	Antigen	Adjuvant	Clinical Phase
VPM 1002 [35]	Live attenuated *Mycobacterium tuberculosis*	-	3
MTBVAC [36]	Live attenuated *M. tuberculosis*	-	2a
Vaccae [37,38]	Heat-killed *M. vaccae*	-	3
MIP [39]	Heat-killed *M. indicus pranii*	-	3
DAR-901 [40]	Heat-killed *M. obuense*	-	2b
RUTI [41]	Cell wall fragments of *M. tuberculosis*	-	2a
M72/AS01 [42]	Protein subunit (Rv1196 and Rv0125)	AS01	2b
H56/IC31 [43]	Protein subunit (ESAT-6, Ag85B, and Rv2660c)	IC31	2b
ID93 + GLA-SE [44]	Protein subunit (Rv2608, Rv3619, Rv3620, and Rv1813)	GLA-SE	2a
GamTBvac [45]	Protein subunit (Ag85A and ESAT-6-CFP10)	CpG ODN	1
TB/Flu-04L[19]	Recombinant influenza vector expressing (Ag85A and ESAT-6)	Flu-04L	2a
ChAdOx1 85A/MVA85 [46]	Recombinant simian adenovirus expressing (Ag85A)	ChAdOx1, MVA	1
Ad5Ag85A [47]	Human adenovirus serotype 5 expressing (Ag85A)	Ad5	1

Whole cell-derived vaccines, subunit vaccines, and viral-vectored vaccines are reported in white, light gray, and dark gray boxes, respectively.

**Table 2:** Structures of PE-like domains in *Mycobacterium tuberculosis* (*Mtb*).

Protein	PDB Code	Chain	Residues	Ligands	Reference
PE25-PPE41 heterodimer	4w4k	C	7–83	None	[84]
	4w4l	A	7–90	EspG5	
PE8-PPE15 heterodimer	5xfs	A	7–84	EspG5	[85]
PE25-PPE41heterodimer	4kxr	A	7–91	EspG5	[83]
PE25-PPE41 heterodimer	2g38	C	8–83	None	[86]

## Supplementary Materials

The following are available online at https://www.mdpi.com/2073-4409/10/1/161/s1: Figure S1: Alignment of the sequence of the C-terminal region of the PGRS domain of PE_PGRS33, PG_II_4, against the entire PGRS region and identification of four PG_II_ sandwich domains.

## Abbreviations

TB	Tuberculosis
WHO	World Health Organization
*Mtb*	*Mycobacterium tuberculosis*
BCG	Bacille Calmette and Guérin
DC	Dendritic cell
IFN	Interferon
TLR	Toll-like receptor
PDB	Protein Data Bank
*Ms*	*Mycobacterium smegmatis*
CDR	Complementarity-determining region
ER	Endoplasmic reticulum
rmsd	root mean square deviations
IL	Interleukin
TNF-α	Tumor necrosis factor–α
