# Supplementary material for: PE_PGRS33, an Important Virulence Factor of Mycobacterium tuberculosis and Potential Target of Host Humoral Immune Response
Source: Cells. 2021 Jan 15;10(1):161. doi: 10.3390/cells10010161 (PMC7830552; doi:10.3390/cells10010161)
Supplement: Supplementary file 1 [file cells-10-00161-s001.pdf]

PE_PGRS33, an Important Virulence Factor of Mycobacterium tuberculosis and Potential Target of Host Humoral Immune Response

Eliza Kramarska [1], Flavia Squeglia [1], Flavio De Maio [2,3], Giovanni Delogu [2,4] and Rita Berisio [1,*]

[1] Institute of Biostructures and Bioimaging, IBB, CNR, 80134 Naples, Italy; eliza.kramarska@gmail.com (E.K.); flavia.squeglia@cnr.it (F.S.)
[2] Dipartimento di Scienze di Laboratorio e Infettivologiche, Fondazione Policlinico Universitario "A. Gemelli", IRCCS, 00168 Rome, Italy; demaioflavio@yahoo.it (F.D.M.); giovanni.delogu@unicatt.it (G.D.)
[3] Dipartimento di Scienze biotecnologiche di base, cliniche intensivologiche e perioperatorie—Sezione di Microbiologia, Università Cattolica del Sacro Cuore, 00168 Rome, Italy
[4] Mater Olbia Hospital, 07026 Olbia, Italy
* Correspondence: rita.berisio@cnr.it

Supplementary Material

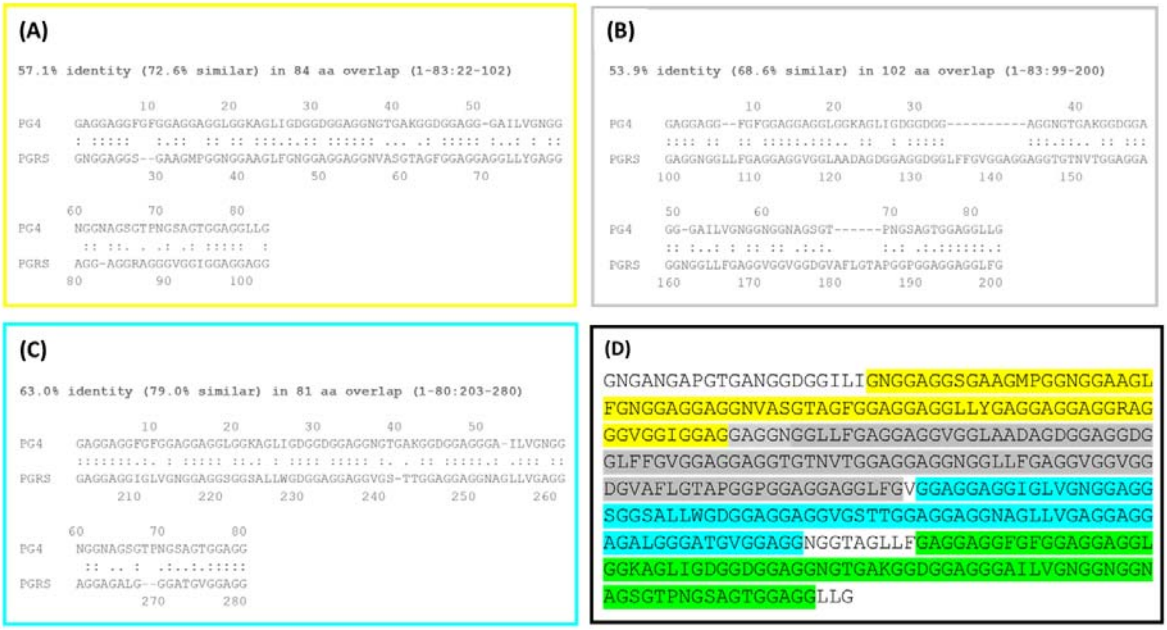

Figure S1. Alignment of the sequence of the C-terminal region of the PGRS domain of PE_PGRS33, PGII4, against the entire PGRS region, using the software LALIGN. Panels A, B, C show alignments of PGII4 with PGII1, PGII2 and PGII3, respectively. Sequence cover of the four domains is given in Panel D.
